# Limb ischemia in peripheral veno-arterial extracorporeal membrane oxygenation: a narrative review of incidence, prevention, monitoring, and treatment

**DOI:** 10.1186/s13054-019-2541-3

**Published:** 2019-07-30

**Authors:** Eleonora Bonicolini, Gennaro Martucci, Jorik Simons, Giuseppe M. Raffa, Cristina Spina, Valeria Lo Coco, Antonio Arcadipane, Michele Pilato, Roberto Lorusso

**Affiliations:** 1 Department of Anesthesia and Intensive Care, IRCCS-ISMETT (Istituto Mediterraneo per i Trapianti e Terapie ad alta Specializzazione), Palermo, Italy; 2 Department of Cardio-Thoracic Surgery, Heart and Vascular Centre, Maastricht University Medical Centre (MUMC), Cardiovascular Research Institute (CARIM), Maastricht University, Maastricht, The Netherlands; 3 Department for the Treatment and Study of Cardiothoracic Diseases and Cardiothoracic Transplantation, IRCCS-ISMETT (Istituto Mediterraneo per i Trapianti e Terapie ad alta Specializzazione), Palermo, Italy; 4 University of Milan-Bicocca, Monza, Italy; 5 Cardiovascular Research Institute Maastricht (CARIM), Maastricht University, Maastricht, The Netherlands; 6 Maastricht University, Maastricht, The Netherlands

**Keywords:** Leg ischemia, Arterial cannulation, ECLS, ECPR, Circulatory support

## Abstract

Veno-arterial extracorporeal membrane oxygenation (V-A ECMO) is an increasingly adopted life-saving mechanical circulatory support for a number of potentially reversible or treatable cardiac diseases. It is also started as a bridge-to-transplantation/ventricular assist device in the case of unrecoverable cardiac or cardio-respiratory illness. In recent years, principally for non-post-cardiotomy shock, peripheral cannulation using the femoral vessels has been the approach of choice because it does not need the chest opening, can be quickly established, can be applied percutaneously, and is less likely to cause bleeding and infections than central cannulation. Peripheral ECMO, however, is characterized by a higher rate of vascular complications. The mechanisms of such adverse events are often multifactorial, including suboptimal arterial perfusion and hemodynamic instability due to the underlying disease, peripheral vascular disease, and placement of cannulas that nearly occlude the vessel. The effect of femoral artery damage and/or significant reduced limb perfusion can be devastating because limb ischemia can lead to compartment syndrome, requiring fasciotomy and, occasionally, even limb amputation, thereby negatively impacting hospital stay, long-term functional outcomes, and survival. Data on this topic are highly fragmentary, and there are no clear-cut recommendations. Accordingly, the strategies adopted to cope with this complication vary a great deal, ranging from preventive placement of antegrade distal perfusion cannulas to rescue interventions and vascular surgery after the complication has manifested.

This review aims to provide a comprehensive overview of limb ischemia during femoral cannulation for VA-ECMO in adults, focusing on incidence, tools for early diagnosis, risk factors, and preventive and treating strategies.

## Background

Veno-arterial extracorporeal membrane oxygenation (V-A ECMO) is an increasingly adopted temporary strategy of circulatory support in cases of refractory cardiac or cardiopulmonary failure, with a constant widening of indications [1–6]. In adults, there are two possible VA-ECMO configurations: central (cV-A ECMO), in which direct cannulation of the right atrium and ascending aorta are obtained, or, more frequently, peripheral (pV-A ECMO), in cases of femoral or axillary vessel cannulation [7]. Central cannulation is more frequently performed in cases of post-cardiotomy shock (PCS), and its reliability in supplying better cerebral and upper body perfusion has to be weighed against an increased number of complications, such as bleeding, infections, and need for transfusions [8–10]. The emergent nature of the shock, as in cardiac arrest scenarios, and the faster and easier accessibility at the bedside, make the peripheral cannulation, and particularly the femoral vessels, the preferred site for percutaneous or surgical cutdown cannula insertion [9, 11]. However, arterial femoral cannulation can cause ipsilateral limb ischemia related to reduced blood flow and oxygen delivery to the distal leg below the insertion point of the cannula, with multiple mechanisms [9, 12–18].

Recent studies have demonstrated that limb ischemia negatively affects patient mortality and survivor’s quality of life [19, 20]. Therefore, early diagnosis and prevention of leg ischemia appear to be of paramount importance [19, 21, 22]. However, clear evidence-based recommendations are still lacking, and the literature on this peculiar V-A ECMO-based aspect is composed primarily of case reports, case series, retrospective cohort studies, and a low number of prospective studies [4, 11, 23]. Depending on the type of cannulation and local protocols, several strategies have been adopted as a preventive approach or rescue treatment of emergent leg ischemia in pV-A ECMO. Moreover, new solutions and devices have become available specifically addressing this ECMO-related shortcoming.

This narrative review of the literature focuses on the incidence, identified risk factors, pathophysiology, monitoring techniques, prevention strategies, and treatment options for distal limb ischemia during pV-A ECMO in order to provide a comprehensive overview of this complicated issue in the era of increasing ECMO support.

## Methods

A literature review was carried through PubMed to identify any study on adults (18 years or older) published from January 2008 to November 2018 to evaluate this condition in the most recent ECMO setting. The terms searched for were “(ECMO OR ECLS) AND (((limb OR leg) AND (ischemia OR hypoperfusion)) OR ((peripheral OR arterial) AND cannulation)).” Only papers published in English were analyzed.

**Fig. 1:**
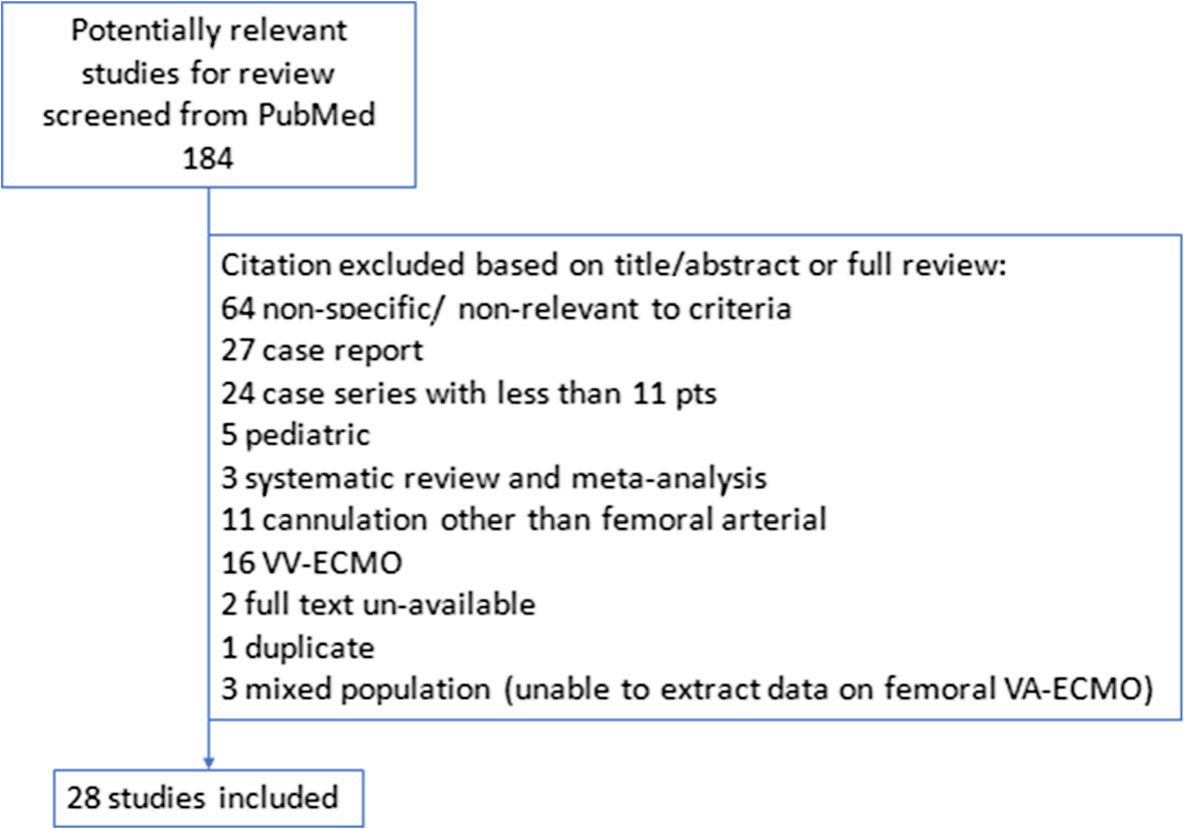
Study selection process

The flow chart of the literature review and screening is shown in Fig. 1. We obtained 184 articles, but only manuscripts including more than 10 patients and reporting cannulation details and leg-related complications for arterial femoral pV-A ECMO were considered for this review. Using a customized form, data were extracted from the 28 remaining articles and stored in an electronic database. Table 1 summarizes the principal findings of the selected articles. Where applicable, the following data were abstracted: study design, number of patients included, age, main comorbidities, percentage of patients with limb ischemia, duration of ECMO run, hospital mortality, cannulation and decannulation strategy, modality and timing for distal perfusion cannula (DPC) placement, and other strategies to prevent or treat limb ischemia.

**Table 1:** Manuscript included for Review

Author, year	Type of study	Patient population	Study endpoint	Main comorbidity	Mean ECMO duration	H survival	Arterial cannula size	Cannulation technique	Decannulation technique	Limb ischemia	DPC timing	DPC size	Ischemia therapy/limb outcome
Sabashnikov, 2018 [24]	R	28 pts. (15 under CPR): 3 (11%) ARDS 1 (3%) DCM 17 (61%) ICM 5 (18%) PAE 1 (3%) MIO 1 (3%) PCS	Primary: Early and mid-term overall cumulative survival (2 years follow-up) Secondary: -Incidences of ECMO-related complications, -Impact of CPR on outcome and changes in hemodynamics -Tissue perfusion factors 24 h after cannulation	NA	96 ± 100 h	11 (40%)	21–23 Fr	PC 27 (90%) SCD 1 (10%)	NA	3 (10%)	Pre-emptive 19 (68%)	6.5 (6.5–8)	Surgical exploration of the femoral artery and embolectomy using a Fogarty catheter.
Park, 2018 [25]	R	255 pts. with HF and/or ARF	Identify risk factors for lower limb ischemia	CAD 83 (32.5%) PVD 5 (2%)	89.8 h	NA (30 days survival 69.8%)	16.5 ± 1.8	PC	NA	24 (9.4%)	Pre-emptive 23 (9%) Rescue 14 (5.5%)	5–7 Fr	2 surgical catheter removal (functional deficit). 14 rescue DPC (Of those, 2 needed surgical intervention and survived with functional deficit.)
Yen, 2018 [14]	R	139 pts.: LI group *n* = 46 No LI group *n* = 93	Identify pre-cannulation variables that are associated with limb ischemia and selection criteria for using DPC for prevention of limb ischemia	No LI group: DM 16 (17%) HT 28 (30%) Uremia 10 (11%) PVD 8 (9%) LI group: DM 10 (22%) HT 17 (37%) Uremia 8 (17%) PVD 11 (24%)	NA	No LI group: 69 (74%) LI group: 25 (54%)	16.5 ± 0.8	PC	NA	46 (33%)	Rescue	6 Fr	NA
Burrell, 2018 [26]	R	144 pts	Complications and outcomes of patients who were commenced on ECMO at a referring hospital compared with patients who had ECMO in a referral center for ECMO.	S 35 (26%) CAD 35 (26%) DM 16 (12%) HF 69 (53%) CT 18 (13%)	7 (4–11) days	105 (72.9%)	17–19 Fr	PC	NA	1 (0.7%)	Pre-emptive	9 Fr	Resolved after DPC insertion at the referral center
Voicu, 2018 [27]	R	46 pts. with refractory CA	Analyze the feasibility and the time interval required for percutaneous cannulation versus anatomic landmark cannulation for va ECMO.	S 21 (46%) DM 5 (11%) HT 17 (37%) HL 15 (33%)	NA	4 (9%)	15–17-19 Fr	PC	NA	0	Pre-emptive	4 Fr	NA
Salna, 2018 [28]	R	192 pts. with CS: 35% AMI 23% PCS 18% ADHF 15% PGD 8.9% other	Incidence of in-hospital lymphocele formation in VA-ECMO patients and identify predictors for its development	DM 65 (33.9%) CKD 52 (27.1% PVD 19 (9.4%)	4 (2–6) days	120 (62.5%)	15–17 Fr	SCD 88 (45.8%)	Surgical	16 (8.3%)	Preventive based on Doppler signal at cannulation	6–10 Fr	NA
Lamb, 2017 [29]	R	91 patients: CS 73 (80%); ARF 14 (15%) PE 3 (4%) VAD failure 1 (1%)	Evaluation of an ischemia prevention protocol	HT 53 (58%) DM 26 (29%) HL 34 (37%) OB 30 (33%) CLD 15 (17%) PVD 6 (7%) CKD 27 (30%)	9 days	38 (42%)	16-24 Fr on pressure-flow curve and pts. size	PC	Surgical	12 (13%) all in patients without preventive DPC	Preventive 55 (60%) Rescue 7 (8%)	5 Fr	DPC 2 (2.2%) DPC+ Fasciotomy 5 (5.5%) Fasciotomy 4 (4.3%)
Pasrija, 2017 [30]	R	20 pts. with PE	Primary outcome: In-hospital and 90-day survival. Secondary outcomes: -Acute kidney injury that required renal replacement therapy -New hemodialysis at discharge -Sepsis, -Tracheostomy, -RV dysfunction at discharge -ECMO-related complications (bleeding that required blood product, stroke after cannulation and vascular complications)	NA	5.1 (3.7–6.7) days	19 (95%)	17–19 Fr	PC	NA	0	Pre-emptive	6 Fr	1 vascular injury due to retrograde type B dissection after ECMO cannulation. Required central cannulation.
Vallabhajosyula, 2016 [31]	R	105 pts. on femoral VA-ECMO: G1 = no DPC G2 = PC DPC G3 = Surgical DPC	Assess if the type of limb perfusion strategy influenced the rate and severity of ipsilateral limb ischemia in peripheral ECLS patients	DM 24 (33%) HT 39 (37%) S 22 (21%)	G1 87.7 ± 119 h G2 88.5 ± 121 h G3 89.2 ± 120 h	G1 21 (60%) G2 14 (61%) G3 32 (68%)	16–20 Fr	NA	NA	G1 7 (20%) G2 6 (26%) G3 1 (2.1%)	Pre-emptive 70 (67%)	7 Fr	4 tromboembolectomy + artery repair 4 fasciotomy 3 cannulation revision 1 amputation
Yeo, 2016 [32]	R	151 pts.: G1 = pre-emptive DPC (44pts) G2 = rescue DPC (107 pts)	Evaluate the efficacy of pre-emptive DPC during ECMO support in term of limb ischemia prevention	DM 25 (16.4%) HT 39 (25.7%) CKD 6 (3.9%) S 27 (17.8%) PVD 11 (7.2%) CVD 5 (3.4%)	G1 4.9 ± 4.9 days G2 6.0 ± 5.4 days	(Overall mortality G1 66 (61.7%) G2 17 (38.6%))	G1 17.2 ± 2.1 Fr G2 17.9 ± 1.8 Fr	PC	NA	10 (6.7%) all in G2	Pre-emptive G1 Rescue G2	5–8 Fr	2 DPC 2 fasciotomy 1 amputation 5 died before therapeutic intervention
Avalli, 2016 [33]	R	100 pts.: G1 with vascular complications 35 (35%) G2 without vascular complications 65 (65%)	Primary endpoint was early vascular complication rate. Secondary endpoint was 1-month and 6-month survival	PVD 8 (8%) CAS 4 (4%) HT 59 (59%) DM 19 (19%) S 25 (25%) HL 20 (20% OB 13 (13%)	G1 5 (3–6) days G2 4.5 (2–9) days	G1 15 (43%) G2 13 (20%)	15–17 Fr	PC	Manual compression 30′ + SafeGuard	34 (34%)	Rescue	7–9 Fr	30 DPC 6 fasciotomy 1 amputation
Tanaka, 2016 [19]	R	84 pts. on pVA-ECMO. 17/84 with vascular complication (G1) 67/84 without vascular complication (G2)	Impact of vascular complications on survival in patients receiving VA ECMO by means of femoral percutaneous cannulation.	S 28 CAD 34 PVD 3 DM24 COPD 10	G1 14.6 ± 6.7 G2 10.6 ± 7.5	G1 3 (18%) G2 32 (48%)	G1 19.8 ± 2.3 G2: 19.7 ± 1.7	PC	Surgical	10 (12%)	Pre-emptive except 7 (41%) G1 10 (15%) G2	NA	Prophylactic fasciotomy
Ma, 2016 [34]	R	70 pts. PCS 44 (63%) ECPR 21 (30%) ARF 5 (7%)	To identify predictive factors for vascular complications, and provide insight into how to reduce these complications	NA	NA	NA	15–24 Fr	44 (63%) SCT 25 (36%) PC 1 not recorded	Surgical	14 (20%)	33 Pre-emptive 6 Rescue	6–8.5 Fr	6 DPC rescue 1 embolectomy 1 fasciotomy 1 embolectomy+ femoral artery repair 1 amputation
Esper, 2015 [35]	R	18 pts. with ACS complicated by CS	Single-center experience	DB 5 (27.8%) HT 9 (50%) HL 2 (11.1%) S 3 (16.7%) PVD 3 (16.7%)	3.2 ± 2.5 days	67%	15–17 FR	PC	NA	4 (22%)	Rescue	NA	DPC
Takayama,2015 [36]	R	101 Group L: (n 51) Group S (n 50)	To compare the clinical outcomes of 2 strategies: conventional approach (using a 15F–24F cannula- Group L) or smaller cannula of15 Fr (Group S)	Group L CAD 22 (43) Ht 26 (51) HL 15 (29) DM 17 (33) COPD 17 (14) Group S CAD 31 (62) HT 33 (66) HL 23 (46) DM 16 (32) COPD 5 (10)	Group L 3.4 (1.0–6.1) days Group S 3.1 (1.9–5.1) days	Group L 31 (61%) Group S 27 (54%)	Group L 17 to 24Fr Group S 15 Fr	Group L PC 22 (43) SCD 29 (57) Group S PC 44 (88) SCD 6 (12)	NA	Group L 2 (4) Group S 2 (4)	Group L 19% Group S 18% Inserted if distal doppler signal is lost	NA	NA
Truby, 2015 [37]	R	179 pts. with CS	Trends in device usage, and analysis of clinical outcomes	CAD 82 (45.8%) HL 72 (40.2%) HT 103 (57.5%) CLD 16 (8.8%) DB 52 (29.1%)	3.58 days	69 (38.6%)	15–23 Fr	NA	NA	25 (13.9%)	9 Rescue	NA	2 Fasciotomy
Saeed, 2014 [38]	R	37 pts.: 25 p VA ECMO	Compare outcome of cECMO versus pECMO patients in the immediate postoperative period.	DM 3 (12%) HT 13 (52%) HL 8 (32%) CAS 3 (12%) CKD 9 (36%) Re-do surgery 5 (20%)	5.8 ± 4.3 days	(30-day mortality 60%)	18–22 Fr	NA	NA	4 (16%)	Pre-emptive	NA	All required surgical intervention
Aziz, 2014 [39]	R	101 pts	Incidence of peripheral vascular complication	HT 33 (32.7%) DM 22 (21.8%) HL 22 (21.8%) S 20 (19.8%)	7.3 days	59 (58.4%)	15–17 Fr	PC	S	8 (8%)	77 (77%) Pre-emptive	NA	8 arterial cannula removal 4 femoral endoarterectomy with patch angioplasty 1 amputation
Papadopoulos, 2014 [40]	R	Total: 360 PCS. 120 (37%) femoral pVA-ECMO	Identification of risk factors for adverse outcome (failed ECLS weaning or in-hospital mortality)	COPD 32 (9%) HT 227 (63%) PH 31 (17%) DM 151 (42%) CVD 22 (6%) PVD 65 (18%) S 122 (34%) CKD 40 (11%)	7 ± 1 days	108 (30%)	NA	Seldinger or 8-mm Dacron Graft	NA	20 (17% of femoral pVA-ECMO)	NA	NA	Fasciotomy 18 (5% of total pts) NA data on femoral pVA-ECMO pts.
Stub, 2014 [41]	SC-POT	26 pts. ECPR (24 cannulated)	Primary outcome: Survival with good neurologic recovery Secondary outcomes: Rates of ROSC, successful weaning from ECMO support and ICU and hospital length of stay.	HT 11 (42%) HL 11 (42%) DM 2 (8%) HF 5 (19%) CAD 4 (15%)	2 (1–5) days	14 (54%)	15 Fr	PC	S	10 (42%)	As soon as possible after ICU admission	8.5 Fr	9 femoral artery repair and surgical placement of DPC 1 fasciotomy
Mohite, 2014 [42]	R	45 pts.: 14 ADHF 8 PCS 6 CS 15 Post CT 2 Bridge to LungT	Compare pts. outcomes focusing on the distal limb perfusion methods (perfusion cannula VS introducer sheat)	NA	Perfusioncannula group: 11.9 ± 9.1 days Introducer sheat group 7.7 ± 4.3	19 (42.2%)	19–21 Fr	20 (44.5%) PC 14 (31%) SCT 11 (24%) Hybrid	NA	9 (20%)	Pre-emptive	Perfusion cannula 10–12 Fr Introducer sheat 6–8 Fr	5 (11.2%) conservative 4 (8.8%) surgery 1 amputation
Spurlock, 2012 [43]	R	On 154 patients (data on 36 patients in PTA-DPC)	Posterior tibial artery for DPC placement	NA	5.8 days	63 (41%)	15–24 Fr on surgeon decision	PC	Direct pressure 30 mins	Available only for PTA-DPC group) 3 (8.3%)	DPC in 68 (44%) PTA-DPC in 36 (24%): 20 (58%) within 6 h of ECMO; 16 (42%) after 6 h of ECMO	6–8 Fr	(Available only for PTA-DPC group) 2 amputation 1 neuropathy
Wong, 2012 [44]	R	20 pts.: 17 (85%) on VA-ECMO	Report single-center experience on cerebral and lower limb NIRS	NA	7 (2–26) days	NA	NA	PC	NA	6 (35%) diagnosed with drop in unilateral lower limb NIRS tracings	Pre-emptive	NA	4 two-compartment prophylactic fasciotomy
Wernly, 2011 [45]	R	51 pts. with Hantavirus cardiopulmonary syndrome	Evaluate the outcome of ECMO support in Hantavirus cardiopulmonary syndrome (HCPS) patients	NA	121.7 h	34 (66.6%)	15–21 Fr	PC 18 (35.3%) SCD 33 (64.7%)	SCD	4 (8%)	Pre-emptive	8–10 Fr	2 thrombectomy, embolectomy, and insertion of an additional cannula in the superficial femoral artery. 2 Amputations
Ganslmeier, 2011 [46]	NA	158 pts	Reviews cannulation strategies and associated vascular complications	NA	3.6 ± 5.2 days	32 (20%)	13–15–17-19 Fr	PC SCT if femoral vessels were small during sonography	Safeguard system	13 (8.2%)	NA	NA	50% Surgical revision and vascular reconstruction 100% prophylactic fasciotomy
Bisdas,2011 [15]	R	143 pts. with ECMO VA	To evaluate such complications to outline basic technical principles for their prevention.	HT 77 (44%) CKD 53 (30%) CAD 47 (27%) COPD 25 (14%) DM 29 (17%) PAD 15 (9%)	6 days (range, 1 to 11 days).	26%	15F or 17F	Percutaneous cannulation in 136 (95%) and by open vessel exposure in 7 (5%).	Manual compression, and femoral compression system	8 pts	Pre-emptive	6F	2 amputation
Foley, 2010 [47]	R	43 pts. on femoral pVAECMO	Examine the outcomes of patients placed on ECMO, including the rate of limb ischemia	NA	NA	NA	Li group 16.9 ± 1.1 No li group 18.0 ± 1.7 Pre-emptive DPC group 17.7 ± 1.8	PC	Surgical	7 (21%)	10 pre-emptive 3 Rescue	NA	4 Decannulation and fasciotomy 3 rescue DPC 1 amputation
Arlt, 2009 [48]	R	13 pts.: 10 (77%) CS 3 (27%) Septic shock	Report 9 years emergency ECMO application	NA	3.5 ± 2.9 days	8 (62%)	15–17 Fr	PC	NA	6 (46%)	Not used	NA	Resolved limb ischemia after cannula switch from the femoral artery to the right subclavian artery.

*Abbreviations*: *ADHF* acute decompensated heart failure, *AF* atrial fibrillation, *AMI* acute myocardial infarction, *ARDS* acute respiratory distress syndrome, *ARF* acute respiratory failure, *CAD* coronary artery disease, *CAS* carotid artery stenosis, *cECMO* centrally inserted ECMO, *CKD* chronic kidney disease, *CO* cardiac output, *COPD* chronic obstructive pulmonary disease, *CPF* cardiopulmonary failure, *CRA* cardiorespiratory arrest, *CS* cardiogenic shock, *CT* cardiac transplantation, *CVD* cerebrovascular disease, *DCM* dilatated cardiomyopathy, *DM* diabetes, *DPC* distal perfusion cannula, *ECMO* extracorporeal membrane oxygenation, *ECPR* extracorporeal membrane oxygenation assisted cardiopulmonary resuscitation, *ESPF* end stage pulmonary fibrosis, *HF* heart failure, *HL* hyperlipidemia, *HT* arterial hypertension, *IABP* intra-aortic balloon pump, *ICM* ischemic cardiomyopathy, *ICU* intensive care unit, *IQR* interquartile range, *LI* limb ischemia, *LungT* lung transplantation, *MIO* myocarditis, *MR* multicenter retrospective, *NA* not available, *NIRS* near-infrared spectroscopy, *OB* obesity, *PC* percutaneous, *PC-DC* percutaneous cannulation and distal perfusion catheter, *PCS* post cardiotomy shock, *PE* pulmonary embolism, *pECMO* peripherally inserted ECMO, *PGD* primary graft disfunction, *PH* pulmonary hypertension, *PPCM* peri-partum cardiomyopathy, *PTA* posterior tibial artery, *PVD* peripheral vascular disease, *R* retrospective, *RHF* right heart failure, *S* smoking history, *SCD* surgical cutdown, *SC-POT* single-center prospective observational trial, *SGP* side-graft perfusion technique, *VAD* ventricular assist device

After a careful evaluation of the literature by two authors (E.B. and G.M.), double-checked by two others (V.L.C. and C.S.), considering the fragmentary data, the different populations mixed in the same studies and the variability of outcomes and interventions, data were considered inadequate to be pooled in a meta-analysis without arriving at potentially erroneous conclusions.

## Narrative review

### Incidence of limb ischemia in pV-A ECMO

Limb ischemia associated with femoral peripheral pV-A ECMO has a reported incidence ranging from 10 to 70% [49, 50]. That highly variable incidence is due to studies performed in populations that are different in baseline characteristics, ECMO indications, cannulation techniques, limb ischemia definition, detection tools, and DPC modalities and timing of insertion [51, 52].

Yang et al., in their large study of major vascular complications in PCS adults receiving femoral–femoral pV-A ECMO support by surgical cutdown, reported a lower incidence of limb ischemia (8.6%), which may be explained in part by the potential advantages of surgically inserted cannulas, with a preventive DPC placement in the majority of the cohort [49]. Nonetheless, in a retrospective series of 84 adult patients on V-A ECMO for cardiac or respiratory failure, Tanaka found a 12% incidence of distal limb ischemia requiring fasciotomy, even in the presence of a prophylactically inserted DPC [19], in line with the findings of Yen et al., who reported that limb ischemia occurred in 33% of patients, even with the use of DPC [14].

With the aim of differentiating the incidence of complications among groups, only two manuscripts can be considered together for cardiogenic shock: one, on 109 patients, reported 16 episodes of limb ischemia (14.7%), 9 fasciotomies (8.3%), and just one case of distal amputation (0.9%) [29, 35].

Three studies dealt with limb ischemia in the ECPR setting: pooling data from these studies on 253 patients, 27 episodes of limb ischemia (10.6%) were detected, though it should be highlighted that in the study by Voicu et al. the mortality was high, and the absence of peripheral complications may be likely related to the marked early mortality [25, 27, 41].

Two studies distinctly considered the concomitant use of V-A ECMO and intra-aortic balloon pump (IABP), describing limb complications. Pooling the data, on 55 patients, we found 4 episodes of limb ischemia (7.2%), with an even protective role for the IABP placement in this setting [25, 31].

Though the comparison of risk of limb complications among the different short-term ventricular assist devices by means of ECMO, Impella, IABP, Tandem heart, is beyond the purpose of this study, this adverse event might be significant when these devices are used in combination as left ventricular (LV) unloading strategy. Recently, Russo et al. reviewed 17 observational studies including 3,997 patients: among them, 1,696 (42%) patients received a concomitant LV unloading strategy while on V-A ECMO, IABP was combined in 91.7% of cases, the Impella percutaneous ventricular assist device in 5.5%, and pulmonary vein or transseptal left atrial cannulation in 2.8%). In this meta-analysis, limb ischemia (RR 1.07; 95% CI 0.90 to 1.27; *p* = 0.47) was not significantly different in patients treated with V-A ECMO associated with another cannulation for left ventricular unloading strategy compared with patients with V-A ECMO support alone [53].

### Pathophysiology and risk factors

**Fig. 2:**
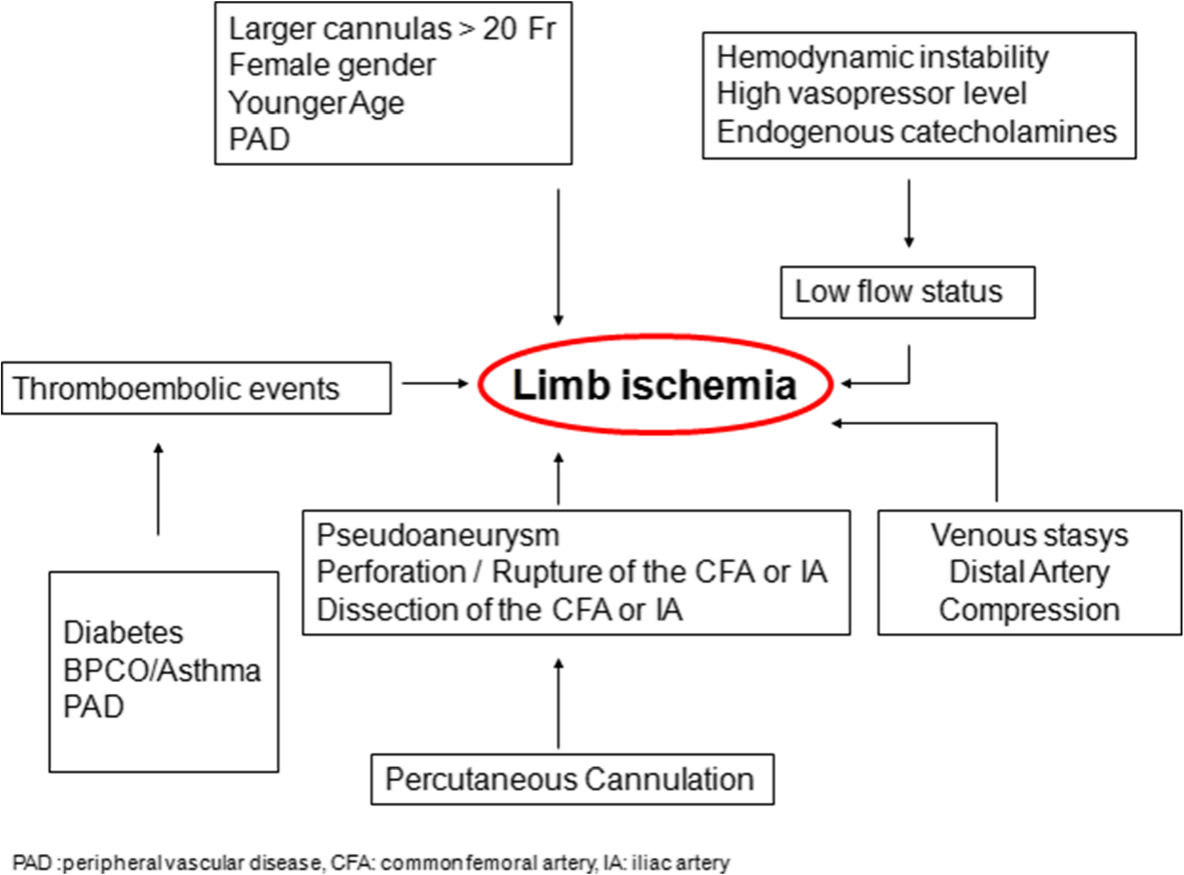
Summary of mechanisms determining leg ischemia during peripheral V-A ECMO run

Limb ischemia in pV-A ECMO patients has a multifactorial genesis that can act at any stage of the ECMO run like at time of cannulation, during support, and at or after decannulation (Fig. 2).

The principal mechanism is a reduced blood flow and related oxygen supply, which arises from an absolute or relative deficit of arterial blood flow to distal tissues. It may be due to a nearly occluding arterial cannula, selective perfusion of the *arteria femoralis profunda*, femoral or iliac vessel damage during cannulation, inadequate peripheral perfusion to match tissue demand, high level of vasopressors, an extrinsic compression of the distal arterial vessel by the same arterial or venous cannula, or atherosclerotic arterial disease, especially in the absence of collateral circulation [14, 15, 19, 25, 54]. Indeed, larger cannulas (> 20 Fr), female gender, younger age, and the presence of peripheral vascular disease are the main risk factors. The use of large cannulas is intuitively associated with limb ischemia due to flow obstruction [54]. However, several studies have not demonstrated such an association, perhaps because the cannula diameter per se is not the cause, but, rather, is the relationship between the cannula and arterial diameter. The catheter/vein ratio frequently adopted in venous cannulation is not widely used in arterial cannulation [55]. A lower incidence of limb ischemia was found when the relationship between body surface area (BSA) and cannula size is greater than 11 [54]. In addition, the cannula may also exert a so-called downstream compression effect, which limits the blood flow below its insertion point [18, 56].

Younger patients, who lack collateral circulation, seem to have smaller femoral arteries, which increase in diameter with age [57]. For the same reason, women have a higher incidence of ischemic complications [19, 29]. Pre-existing atherosclerotic disease can increase the risk of plaque dislodgment and embolism during both cannulation and decannulation, as well as increase the technical complexity of the procedure, with higher risk of dissection, or significantly reduce the antegrade flow [15, 19, 58]. Moreover, an increased venous pressure, with consequent reduced perfusion pressure, may contribute to tissue hypoxia [56]. Furthermore, arterial compression, distally to the cannulation site, may be induced also by an incorrect lateral course of the venous cannula.

Considering the comorbidities, diabetes and respiratory diseases are independent risk-factors for limb ischemia during pV-A ECMO. Diabetes is characterized by a proinflammatory state, with macro- and micro-vascular alterations that can exacerbate limb hypoperfusion during a low flow state [12, 59]. Pulmonary diseases, such as asthma and chronic obstructive pulmonary disease (COPD), are characterized by a state of chronic hypoxia, which induces endothelial damage, inflammatory state, and development of atherosclerotic disease [60].

Danial and colleagues found limb ischemia independently associated with the SOFA score at ECMO cannulation, suggesting that the patient’s condition (and a proinflammatory state), namely the compensatory capacity for peripheral hypoperfusion, may be more relevant than the single mechanical procedure [61].

Limb ischemia does not account for only local vascular damages. The persistence of prolonged ischemia can lead to an irreversible damage of the leg, with the most severe cases complicated by compartment syndrome, eventually requiring fasciotomy or even limb amputation [62]. Furthermore, reperfusion of the ischemic limb by re-establishing or enhancing distal flow may represent an additional threat because of proinflammatory and wasting mediators released into the systemic circulation, causing rhabdomyolysis, systemic inflammatory state, and multi-organ dysfunction [63, 64].

### Diagnosis

The Intersociety Consensus for the Management of Peripheral Arterial Disease (TASC II) defines acute limb ischemia as a sudden decrease in limb perfusion that causes a potential threat to limb viability [65]. The latest AHA/ACC guidelines include a specific section on limb ischemia during hemodynamic support and called “Asymptomatic Artery Disease,” the obstructive disease in patients who require large-diameter catheter access for life-saving procedures [66]. Diagnostic tools for early diagnosis are summarized in Table 2.

**Table 2:** Summary of diagnostic tools for early detection of limb ischemia during V-A ECMO

	Every hour	Every shift	Altered perfusion
Bedside nurse	Bilateral clinical evaluation	Doppler pulse check	Doppler pulse check
	Temperature		
	Appearance		
	Refilling Time		
ECMO specialist		Bilateral clinical evaluation	Bilateral clinical evaluation
		ECMO flow check	ECMO flow Check
		Vasopressor balance	Vasopressor balance
		DPC flow check	DPC flow check
NIRS	NIRS	NIRS	NIRS
Radiologist			ECHO Doppler
			Angiography

Monitoring distal perfusion in pV-A ECMO is of paramount importance in order to timely detect and treat ischemia, with favorable limb and patient outcomes. As in other acute conditions, “time is tissue,” but, nevertheless, there is no standard of care regarding monitoring. Several tools have been adopted, and they can be grouped into clinical examination, the extensive use of ultrasound and Doppler ultrasonography and, recently, the use of near-infrared spectroscopy (NIRS) as a surrogate for distal perfusion. As a general rule, during pV-A ECMO, any suspicion of limb ischemia should conduct to an increase in monitoring to reach a complete diagnosis: clinical examination should be followed by Doppler sonography and eventually leading to angiography and complete involvement of a multidisciplinary team.

#### Clinical signs and diagnostic tools

The clinical pattern of acute limb ischemia was described by Pratt, in 1954, as the 6 Ps signs: paleness, pulselessness, paraesthesia, paralysis, pain, and poikilothermia [67]. Clinical evaluation should be routinely performed several times per shift [68]. High level of suspicion for ischemia can arise from skin temperature (cold), appearance (pale, mottling), compared with the contralateral limb, and refilling time [42].

Guidelines recommend ultrasound (US)-guided vascular access in order to reduce immediate and late complications [55, 69]. US can be useful in pV-A ECMO at the time of cannulation in order to select the optimal cannulation site, avoiding atherosclerotic arteries, sparing the deep femoral artery origin with its collateral flow to the limb and, finally, providing information regarding vessel size and measurement to guide cannula selection and implantation. First-pass success and reduced groin hematoma rates have been described when US-guided vascular access is compared with landmark techniques [70, 71]. No studies have investigated the relationship between the common femoral artery and cannula diameter in determining leg ischemia [54]. During pV-A ECMO support, if Doppler flow is audible, distal limb perfusion pressure can be evaluated by placing a sphygmomanometric cuff at the ankle just proximal to the Doppler probe. A perfusion pressure of less than 50 mmHg indicates limb ischemia [72]. Moreover, Doppler ultrasonography (D-US) can be used to monitor peak systolic velocity (PSV) of distal arteries, such as the posterior tibial or dorsalis pedis. Feasibility of Doppler-derived flow velocity in pV-A ECMO patients as a monitoring tool for leg ischemia has been reported by Breeding et al. [73]. However, PSV was positively correlated with pulse pressure and negatively with ECMO pump flow, making its usefulness unclear in fully supported ECMO patients.

NIRS use is increasing in adult anesthesia and critical care [74]. It employs light of near-infrared wavelengths (700–1000 nm) emitted and detected by a probe applied to a body region. Differently from a pulse oximeter, NIRS monitors the difference between oxy- and deoxygenated hemoglobin (HBO-HBD), and a pulsatile blood flow is not a prerequisite for its functioning. HBO-HBD reflects oxygen uptake in the tissue bed and is defined as regional oxygen saturation (rSO_2_) [75]. Because of the independence of pulsatile blood flow, rSO_2_ comprises arterial and venous contribution, the latter being the most important [76].

Wong et al. first described NIRS in ECMO patients to concomitantly monitor both cerebral and limb perfusion [77]. They included NIRS monitoring into the treatment protocol and identified clinically significant events that warranted intervention when rSO2 dropped below 40 or more than 25% from baseline [44, 78]. More recently, NIRS monitoring in both cannulated and non-cannulated leg in pVA-ECMO patients has been used to differentiate between cannula-related obstruction (delta-rSO_2_ between cannulated and non-cannulated leg < 15%) and other causes of hypoperfusion [77]. All patients with clinical evidence of limb ischemia had rSO_2_ below 50% for longer than 4 min, and a positive predictive value of 86% was calculated [77].

### Limb ischemia prevention

Many prevention strategies have been proposed to avoid limb ischemia in pV-A ECMO patients: cannula size and cannulation side selection, cannulation technique, and placement of a smaller cannula for anterograde or retrograde (ankle) distal perfusion [79].

**Fig. 3:**
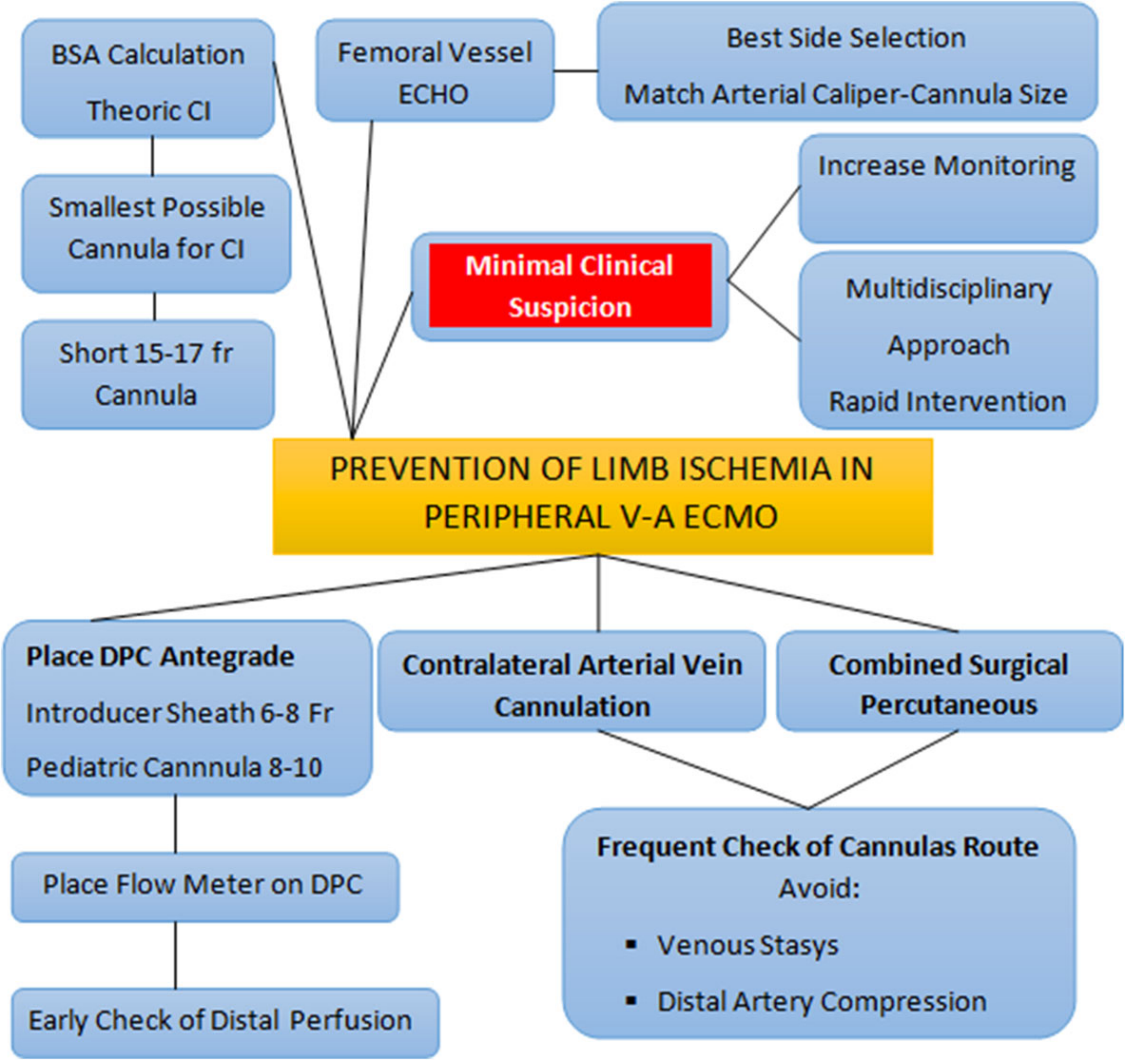
Proposed flow chart illustrating strategies for limb ischemia prevention

A summary of proposed preventive strategy is illustrated in Fig. 3.

#### General considerations for arterial cannula selection

The selection of type and size of the arterial cannula should be based on a balance between the targeted flow rate and anatomical considerations. Generally, the first consideration starts from the evaluation of the patient’s BSA and, conventionally, cannulas are chosen to obtain a flow equivalent to a cardiac index (CI) of 2.2–2.5 L/m^2^/min [80].

This accepted rule should be considered as the starting of the decision making process since it is challenged by the fact that the main determinant of the ECMO flow is the capacity of the drainage cannula (determined by the size, the number of side holes and the position—preferred in the right atrium), and that generating a full flow is not always necessary during V-A ECMO. In some cases, it is even detrimental when the peripheral inflow determines an excessive increase in the ventricular afterload, with consequent left ventricular distension [9].

In this light, combining the targeted flow, the US-doppler of the femoral arteries, and - in case of surgical cut - also the inspection and palpation, the smallest possible cannula should be preferred. Moreover, the arterial cannulas are also shorter to provide less resistance to the flow. According to the center’s strategy for cannulation and flow support, the arterial cannulas usually range from 15 Fr to 23 Fr. Takayama et al. have documented a protocol of using a small size cannula, 15 Fr diameter, with promising results of comparable clinical support, but a lower rate of complications [36].

#### Cannulation technique

Arterial and venous cannulation can be achieved with a surgical cutdown, or a percutaneous approach. In the open technique, a surgical exposure of the femoral vessels can be obtained by a longitudinal or transverse skin incision of the groin and dissection of subcutaneous tissue and fascia. Identification of ligament, common femoral artery, and the bifurcation are important in detecting the proper cannulation site. Inspection and palpation of vessels contribute to an adequate cannula-size selection and avoid dangerous calcifications. A 4/0 or 5/0 polypropylene purse-string is then performed on the vessel. The purse-string should be in the longitudinal direction, and as small as possible, in order to avoid stenosis of the artery after cannula removal and purse-string knotting. The venous and arterial cannulas are placed using a modified Seldinger technique. The venous cannulation should be performed first, followed by the arterial cannulation because of the anatomic relationship and course of the vein compared to the artery. Alternatively, after distal and proximal vascular clamp placement, a transversal incision is made on the artery and the cannula is gently introduced. In these circumstances, longer vessel isolation is advisable, with a vessel loop placement around the vessel to achieve better control during the cannula implantation. Purse-strings are tightened around the vessel entry and secured to the cannula by snuggers long enough to allow sufficient prolene length for final knotting at cannula removal. The plastic snuggers are looped and hidden in the groin pouch. In the so-called pseudo-percutaneous approach, the femoral vessels are exposed with an open approach, but the cannulas are tunneled through two separated small incisions at 3–4 cm distally from the groin vessel exposure, allowing complete closure of the femoral incision [18]. Further justification for such an approach is the reduced risk of bleeding and infections post-ECMO implantation, easier nursing care, and easier device removal, though it still requires an open surgical closure allowing better control of the vascular entry site and embolectomy in case of distal or proximal clots. Femoral artery perfusion can be also achieved through a Dacron or Hemashield prosthetic graft (6–8 mm) anastomosed end-to-side onto the femoral artery, thus maintaining antegrade as well as retrograde arterial flow to the ipsilateral lower limb [81–84]. This approach is aimed at establishing the flow through a small femoral artery, and simplifying the decannulation procedure. However, excessive arterial flow to the limb and reduced flow to the rest of the body can occur. A distal venous draining catheter connected to the ECMO venous cannula may be needed in order to limit limb edema [17, 83].

The percutaneous cannulation technique is performed using the Seldinger technique under ultrasound guidance and, whenever available, transesophageal echocardiography can guide the entire procedure, detecting the location of the guidewire and any new or increasing pericardial collection [85, 86]. After ultrasound identification, femoral artery is cannulated using a percutaneous kit, avoiding a lateral or backwall puncture rather than to achieve a front-wall puncture. The wire is advanced to the abdominal aorta under fluoroscopic or transesophageal guidance, whenever available, and, after dilation, the cannula is introduced.

Compared with percutaneous cannulation, surgical cannulation is adopted mainly in PCS, and associated with fewer vascular complications [87]. A propensity-score-matched study explored the differences in the rate of limb ischemia at the same center between the percutaneous and surgical approaches and found no significant difference, though the trend was in favor of the percutaneous approach [61]. Recently, Deschaka et al. described a hybrid V-A ECMO configuration in which the ascending aorta was cannulated via an 8 mm prosthesis directed subxyphoidally, and the femoral vein was percutaneously cannulated in order to limit limb ischemia due to the femoral artery cannulation, at the same time avoiding the risks of an open thorax [88]. Saeed et al. adopted a similar approach in 9 cases of PCS, demonstrating its feasibility [89].

#### Cannulation site selection

The puncture of the femoral artery can be performed ipsilaterally or contralaterally according to the center and the surgeon’s preference [18]. The most appropriate site for pV-A ECMO cannulation has not been well identified, but bilateral groin cannulation (one cannula in one groin, the other in the contralateral one) might be preferable due to the reduction of vessel compression and the avoidance of the association of reduced perfusion flow and venous congestion [90, 91].

#### Distal perfusion cannula (DPC)

The Extracorporeal Life Support Organization (ELSO) guidelines state that “if distal arterial flow to the leg is inadequate a separate perfusion line is placed in the distal superficial femoral artery by direct cutdown, or in the posterior tibial artery for retrograde perfusion.”

The most adopted preventive strategy is the placement of a DPC in the proximal superficial femoral artery. The insertion of the DPC can be performed percutaneously with ultrasound or fluoroscopy guidance, and in this case, the wire for antegrade distal perfusion cannula should be placed at the time of main femoral cannula placement, based on a better exposure and puncture without the proximal cannula in place. In the case of surgical cutdown, it can be performed either by surgical arteriotomy or by direct vision with a modified Seldinger technique, and in a recent meta-analysis, the limb ischemia was lower with DPC placement by open access [92].

There is a significant variability of the DPC type and caliper among centers. This catheter is usually connected to the side port of the arterial cannula using a 6-in. extension tubing with an intervening three-way stopcock for a regular check of the flow and eventual line to administer arterial vasodilator. DPCs are reported in sizes from 5 to 14 Fr; the most adopted are central venous catheter and vascular introducer sheaths (usually 6–8 Fr) [93].

**Fig. 4:**
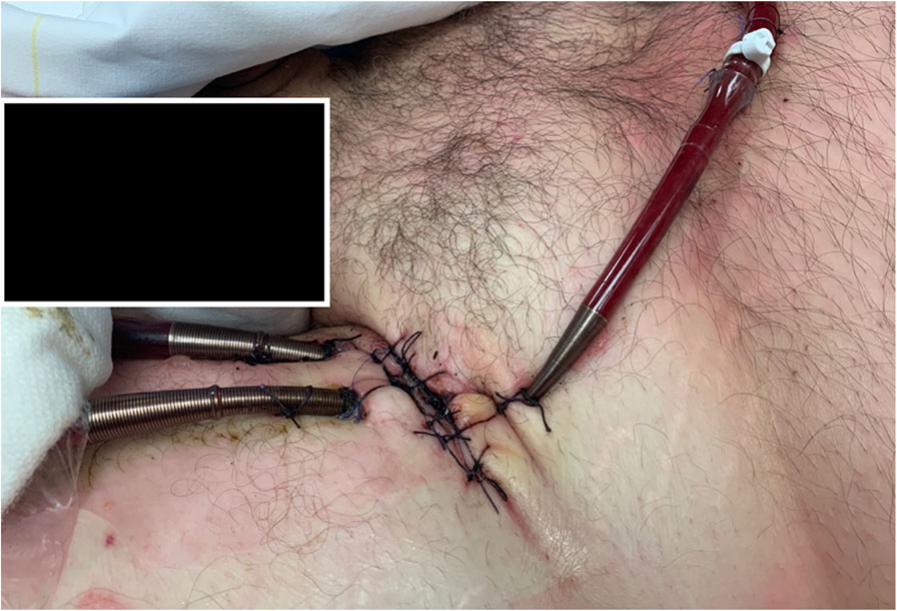
Possible contralateral cannulation during V-A ECMO: bi-groin cannulation with combined surgical/percutaneous approach. The distal perfusion cannula is a pediatric 10 Fr cannula connected without a stopcock to the side port of the femoral cannula. (Original photo provided by R.L.)

The use of pediatric armed arterial cannulas (8 or 10 Fr) are also reported (illustrated in Fig. 4), and there are likely some advantages, such as direct connection between the shunt line and the DPC, avoiding a stopcock, and allowing a better configuration in terms of flow patterns and preventing dangerous kinking. This was investigated by Mohite et al. showing lower limb ischemia comparing to the use of the introducer sheath [42]. Rao et al. reported a case of DPC insertion from the contralateral femoral artery and angiographically guided to restore perfusion of both the superficial and profound femoral artery of the cannulated leg [94]. A relevant trick with this technique is to place a flow meter also on the DPC applied along the DPC circuit to recognize the effective distal flow and counteract if the flow is reduced but also partially reducing the flow by a clamp in case of excessive distal flow.

#### Bidirectional cannula

Recently, to overcome the distal limb ischemia, a new bidirectional femoral arterial cannula (LivaNova PLC, Arvada, CO, USA) has been proposed and tested during cardiopulmonary bypass (CPB) in 15 patients [95]. This cannula, similar to a standard femoral arterial cannula, has a 120-degree angled elbow with a side hole for antegrade perfusion to alleviate the compression of the femoral artery below the insertion point. The external diameter of the distal section of the 19 Fr bidirectional cannula is 7 mm, and the external diameter, obliquely at the cannula elbow, is 8.4 mm. This cannula showed appropriate bypass flows in the extracorporeal circuit, satisfactory line pressures, mean arterial pressures adequate to provide organ perfusion, and allowed an adequate distal flow in 14 of 15 patients checked with NIRS, with no ischemic complications [95]. A study using a percutaneous insertion technique of the femoral bidirectional cannula in patients requiring V-A ECMO is currently in progress, and the promising results in the short-term support of CPB, if confirmed in longer support for V-A ECMO, may offer the community a relevant technological improvement for clinical use in a number of different perfusion settings.

### ECMO weaning and decannulation strategy

Danial et al. found higher rates of vascular complications after decannulation in a percutaneous group compared with a surgical cutdown group (14.7% vs. 3.4%, *p* < 0.01) [61]. This result is rarely highlighted, and not confirmed by the available literature. Surgical closure at decannulation may enhance safer decannulation, with reduced bleeding, pseudoaneurysm formation, compression time likely associated with local thrombosis, check for distal flow, and allow repair in case of vessel damage or structural impairment [61]. It is indeed advisable to perform an immediate control after cannula withdrawal of the distal artery pulsatility and of the presence of flow at distal leg portion since embolization, when it does occur, is usually observed just after decannulation.

Weaning trial of V-A ECMO will also decrease flow through the distal perfusion cannula such that ischemia may result from a prolonged duration of low ECMO flow despite the presence of a distal perfusion catheter; consequently, in patients with critical limb perfusion, the length of weaning trial should be reduced [58].

A new method of percutaneous arterial closure proposed recently is the use of specific closure devices, usually imported into the V-A ECMO practice from the interventional cardiology environment. These devices have been used for closure in case either of percutaneous or surgical approach and seems able to reduce bleeding and surgical site infections, but are challenged by the need of expert users who are not always involved in V-A ECMO management. Their use is still restricted to some centers and documented in short reports. Majunke et al. proposed the combined use of the Perclose ProGlide system (Abbott Vascular) and the AngioSeal device (St. Jude Medical), while Montero-Cabezas et al. reported the use of the MANTA vascular closure device (Essential Medical Inc., Malvern, PA) [96, 97]. Further prospective-focused studies should explore this field in order to understand the feasibility of such an approach.

### Treatment

The key to deciding the treatment of limb ischemia during V-A ECMO is to distinguish a threatened from a nonviable extremity, bearing in mind that the determination of whether ischemia is reversible is rather subjective (largely based on appearance of soft tissue and amount of necrotic tissue). Often, it can be determined only after conservative management has failed, but the longer the symptoms are present, the less likely the possibility of limb salvage.

According to the Society of Vascular Surgery standard, the loss of the Doppler arterial signal indicates that the limb is threatened (stage II). The absence of both arterial and venous Doppler signal indicates that the limb may be irreversibly damaged and non-salvageable (stage III) [72].

Limb ischemia in femoral cannulation ECMO is largely transient and completely reversible with the removal of the cannula or the insertion of DPC. In a small percentage of patients, it is irreversible, with refractory muscle damage eventually leading to leg amputation (up to 14% of cases) or even contributing to patient death. When the ischemia is considered irreversible the potential amputation should not be delayed since tissues necrosis may extend with higher risk of sepsis, bleeding, intractable acidosis and systemic release of toxic mediators.

Acute compartment syndrome (ACS) is a severe clinical condition caused by increased tissue pressure, inducing a reduction of the perfusion, with consequent further ischemia. It can lead to severe functional impairment due to muscular necrosis and neurological damage, or to ischemic muscle shrinking, with consequent limb deformity.

When limb ischemia is ongoing, a thorough evaluation should be constantly performed to balance between the need for adequate systemic flow, vasopressors use, and the risk associated with further surgical procedures that are in any case at risk of bleeding and further vascular damage in VA-ECMO patients [98]. Consequently, first, the amount of vasopressor should be considered and eventually reduced or discontinued, also optimizing volemia and oxygen transport by hemoglobin. In case of mild reduced perfusion, optimizing peripheral temperature is a general adopted care, while the administration of peripheral vasodilator through the DPC may help in diagnosing the reversibility of reduced perfusion due to excessive vasoconstriction. Moreover, during limb ischemia, in the absence of bleeding, anticoagulation should be kept at the highest level according to the center’s range.

The invasive therapies include removal and repositioning of the cannula (contralateral limb, subclavian, or aortic cannulation) and repair of the artery with suture and/or bovine pericardial patch angioplasty, Fogarty catheter-based embolectomy, limb fasciotomy or amputation [19, 29, 47].

Yau et al. found that in their cohort of 34 patients with limb ischemia after V-A ECMO, 3 required lower extremity amputation, and 7 needed fasciotomy for a compartment syndrome [12, 15, 19]. Moreover, Tanaka reported an independent association between major vascular complications and mortality in 84 patients on V-A ECMO, with 20% experiencing major ischemic injury, and 12% requiring fasciotomies [19].

Endovascular methods, including balloon angioplasty or stenting, can be additional options. In these circumstances, open reconstruction of the femoral vessels with endarterectomy and patch angioplasty or femoral-femoral bypass grafting can help to improve the arterial flow.

**Fig. 5:**
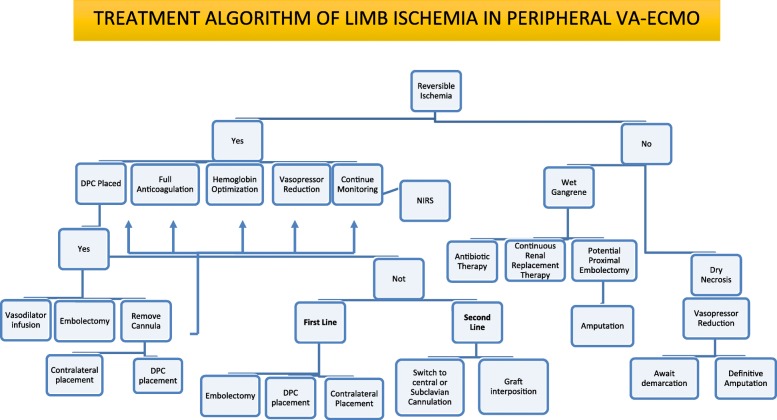
Proposed flow chart for the treatment of limb ischemia in V-A ECMO

A proposed flow chart for limb ischemia treatment considering general clinical and surgical approach is shown in Fig. 5.

## Conclusion

V-A ECMO is a life-saving procedure that provides mechanical circulatory support for advanced heart failure. Advances in technology, portability, and easy-to-use devices have led to its use worldwide, even outside the cardiac surgery setting, with a progressive improvement in survival.

In cases of peripheral cannulation, limb ischemia is still frequent, particularly if preventive strategies are not adopted, and the consequences of this complication can impact negatively on the survival or the long-term functional outcomes.

A strict monitoring protocol for early detection and timely interventional strategy to guarantee an adequate peripheral flow restauration are mandatory to reduce the incidence and improve the prognosis and outcome of the V-A ECMO patient.

V-A ECMO is a complex, resource-intense, and high-risk type of mechanical support. Future research should focus on complications, providing more clues as to the effectiveness of different preventive and therapeutic strategies to guide a further increase in survival.

## Data Availability

The datasets used and analyzed are available from the corresponding author on reasonable request.

## Abbreviations

BSA: Body surface area
CPB: Cardiopulmonary bypass
cV-A ECMO: Central veno-arterial extracorporeal membrane oxygenation
DPC: Distal perfusion cannula
PCS: Post cardiotomy shock
pV-A ECMO: Peripheral veno-arterial extracorporeal membrane oxygenation
V-A ECMO: Veno-arterial extracorporeal membrane oxygenation
